# Actinomycetes: The Antibiotics Producers

**DOI:** 10.3390/antibiotics8030105

**Published:** 2019-07-29

**Authors:** Yvonne Mast, Evi Stegmann

**Affiliations:** 1 Department of Bioresources for Bioeconomy and Health Research, Leibniz Institute DSMZ-German Culture Collection for Microorganisms and Cell Cultures, 38124 Braunschweig, Germany; 2 Institute for Microbiology, Technical University of Braunschweig, 38106 Braunschweig, Germany; 3 Department of Microbiology/Biotechnology, Interfaculty Institute of Microbiology and Infection Medicine, Faculty of Science, Eberhard Karls University of Tübingen, Auf der Morgenstelle 28, D-72076 Tübingen, Germany; 4 German Center for Infection Research (DZIF), Partner Site Tübingen, D-72076 Tübingen, Germany

Actinomycetes are well known as an inexhaustible source for antibiotics. Most of the known antimicrobials today were originally isolated from actinomycetes, especially from the genus *Streptomyces*. The produced substances include all important drug classes used in clinics today, such as β-lactams, tetracyclines, macrolides, aminoglycosides, or glycopeptides. However, in the past years the effectiveness of these impressive weapons have become endangered by the rise of resistances of live-threatening pathogenic bacteria. In addition to that, the golden era of antibiotic discovery has been over for quite a while and the antibiotic pipeline was feared to run dry. Next-generation sequencing techniques in combination with genome mining approaches revolutionized the field of antibiotic research and might reboot the pipeline in the near future. In 2002, the first *Streptomyces* genome sequence was published [1]. This was the genome sequence of the model actinomycete *Streptomyces coelicolor*. Mining this sequence revealed that *S. coelicolor* harbors 22 secondary metabolite gene clusters but indeed produces only four of the encoded metabolites under standard lab conditions. Currently, more than 625 genome sequences are available only of the genus *Streptomyces* [2]. Genome mining analyses suggest that less than 10% of the genetic potential of antibiotic producers is currently being used [3], which implicates that there is a huge untapped genetic reservoir waiting to be exploited for drug discovery. In addition to that, metagenomic data indicate that there are far more potential antibiotic producers in nature awaiting isolation and investigation. Thus, today, almost 80 years after Selman Waksman introduced the genus of *Streptomyces* and, with actinomycin being the first antibiotic that was isolated from an actinomycete, these bacteria still represent a treasure chest for the identification of novel antibiotics. 

Novel cultivation strategies, elaborated screening techniques, new genetic manipulation tools, further insights into physiological aspects of actinobacterial live style, and also knowledge on new secondary metabolite biosynthetic pathways may open up a new era of antibiotic drug discovery. 

In the Special Issue of "Actinomycetes: The Antibiotics Producers" we will highlight the latest research findings in the field. The current issue brings together 19 publications (9 research articles and 10 reviews) from expert groups, which address scientific topics associated with the biology of actinomycetes as well as their antibiotic biosynthesis capacity.

Two research articles deal with the isolation and characterization of actinobacteria from unique habitats. Using a high-throughput sequencing approach, Maciejewska et al. were able to show that there is a quite complex actinobacterial community present in cave moonmilk deposits obtained from the cave “Grotte des Collemboles”, Belgium [4]. By applying several strategies to allow growth and isolation of hard-to-culture actinobacteria, Adam et al. were able to isolate new actinobacterial representatives from these cave moonmilk deposits and demonstrated that some of them exert antibacterial activity [5]. Another extraordinary source for novel actinobacteria producing unique chemistry are certain insects. Benndorf et al. isolated 97 actinobacteria from the fungus-growing termite *Macrotermes natalensis* and analyzed them in terms of their antibiotic production properties. Thereby, the authors isolated several new actinobacterial species and identified a dichlorinated diketopiperazine derivative and two tetracyclic lanthipeptides (rubrominins A and B) as new secondary metabolites [6]. However, not just new strain isolates but also old strain isolates can be promising sources for novel natural compounds, when investigated with state-of the art analytical technologies. This has been effectively highlighted in the review of Takahashi and Nakashima, which gives an update on novel compounds from old strains of the Kitasato Microbial Library [7]. Indeed, the authors describe the identification of 36 novel compounds from 11 actinomycetes, most of them more than 35 years old. Thereby, the list of producers resembles a quite diverse set of actinomycetes, which illustrates that also non-*Streptomyces* actinomycetes represent good sources for novel natural compounds. This is also underpinned by the study of Iorio et al., which specifically focusses on the underexplored genus *Actinoallumurus*, for which two novel polyether substances (α-770 and α-823) were described [8]. This raises the question if and to what extent gene cluster composition differs between different types of actinobacterial antibiotic producers. Two of the Journal’s papers deal with that issue in more detail: Choudoir et al. and Vicente et al. focus on comparative biosynthetic gene cluster (BGC) distributions in similar streptomycetes and thereby concentrate on the evolutionary dynamics of BGCs within these organisms [9,10]. These manuscripts already depict the importance of omics approaches in antibiotic research, which in general is shortly highlighted in the review of Genilloud [11]. Finally, a very comprehensive review on novel strategies and innovative methods to get access to novel natural compounds from actinomycetes is given by Hug et al., which also offers a very reasonable outlook to future perspectives in antibiotic research [12]. 

Besides the studies that aim to identify novel antibiotics, the Special Issue also contains four papers dealing with specific antibiotics and their biosyntheses: Schwarzer et al. report on the biosynthesis of the polyketide rishirilide B [13], whereas Hofeditz et al. describe the production and structure elucidation of the novel polyphenolic tridecaketide lysoquinone-TH1 [14]. Both compounds are polyketides, and biosynthesis has successful been made possible after heterologous expression of the respective gene clusters in *Streptomyces albus*. Viana Marques et al. provide an update on β-lactam inhibitors and their applications [15], whereas Musiol-Kroll and Wohlleben focus on the biochemical function and usage of acyltransferases during polyketide biosynthesis for engineering approaches [16]. 

Especially when it comes to engineering and production attempts of the respective compounds, regulation and self-resistance of the producer organisms are important factors to be targeted in order to increase yields. Thus, aspects of regulation and self-resistance are also addressed in the Special Issue: Ordóñez-Robles et al. summarize the current knowledge on regulation of tacrolimus biosynthesis in *Streptomyces tsukubaensis* [17]. Alduina et al. report on the regulatory network influencing the biosynthesis of the glycopeptide antibiotic A40926 from *Nonomuraea* sp. [18], whereas Binda et al. concentrate on the self-resistance mechanisms and characteristics of glycopeptide producers in general [19].

Besides their overwhelming importance as antibiotic producers, actinomycetes undoubtedly have further imposing characteristics, and some of them are handled in the Special Issue. For example, actinomycetes undergo a complex life cycle, whereby morphological development and secondary metabolism often coincide. Manteca and Yagüe review strategies to improve *Streptomyces* differentiation in liquid cultures with the aim to elicit antibiotic production [20]. Furthermore, actinomycetes are well known as potent producers of extracellular hydrolytic enzymes, which is relevant for industrial applications. Here, Gullón and Mellado give an overview focusing on the secretion machinery of the most widely used host strains for exoenzyme production—*Streptomyces lividans* [21]. Finally, Jones reports on known and novel aspects of the polynucleotide phosphorylase (PNPase) in *Streptomyces*, which exerts a dual function as 3′-5′-exoribonuclease and RNA 3′-polyribonucleotide polymerase [22]. 

We are happy to bring together so many valuable works from respected experts in the field. It was our great pleasure to be Guest Editors of this special issue of *Antibiotics.*
